# Factors Important for Work Participation Among Older Workers with Depression, Cardiovascular Disease, and Osteoarthritis: A Mixed Method Study

**DOI:** 10.1007/s10926-015-9597-y

**Published:** 2015-07-26

**Authors:** Cécile R. L. Boot, Anja Th. C. M. de Kruif, William S. Shaw, Allard J. van der Beek, Dorly J. Deeg, Tineke Abma

**Affiliations:** Department of Public and Occupational Health, EMGO Institute for Health and Care Research, VU University Medical Center Amsterdam, PO Box 7057, 1007 MB Amsterdam, The Netherlands; Body@Work, Research Center on Physical Activity, Work and Health, Amsterdam, The Netherlands; Department of Metamedica/Medical Humanities, EMGO Institute for Health and Care Research, VU University Medical Center Amsterdam, Amsterdam, The Netherlands; Department of Health Sciences, EMGO Institute for Health and Care Research, VU University Amsterdam, Amsterdam, The Netherlands; Liberty Mutual Research Institute for Safety, Hopkinton, MA USA; University of Massachusetts Medical School, Worcester, MA USA; Department of Epidemiology and Biostatistics/Longitudinal Aging Study Amsterdam, EMGO Institute for Health and Care Research, VU University Medical Center Amsterdam, Amsterdam, The Netherlands

**Keywords:** Longitudinal studies, Employment, Chronic disease, Social participation

## Abstract

*Purpose* The aim of this study was to gain insight into differences and similarities in factors important for work participation in older (58–65 years) workers among three different chronic diseases: depression (D), cardiovascular disease (C), and osteoarthritis (O). *Methods* A mixed method design was used, with a qualitative part (in-depth interviews) with 14 patients with D, C or O and a quantitative part based on the 2002–2003 cohort of the Longitudinal Aging Study Amsterdam. We analysed and compared 3-year (response 93 %) predictors of paid work in 239 participants with D, C, or O using regression analyses. The qualitative findings were integrated with the quantitative findings aiming at complementarity. *Results* Common factors important for work participation were: working at baseline; male gender; lower age; partner with paid work; better physical and mental health; and higher mastery scores. The qualitative analyses added autonomy in work and provided contextual information regarding the perceived importance of working as factors important for participation in paid work. For D and C, work gave purpose in life and enhanced social contacts. Participation in work was perceived as necessary to structure life only for D. *Conclusion* Most factors important for work participation were similar for D, C, and O. However, the interviews revealed that for D, the context and the meaning attributed to these factors differed.

## Introduction

The ageing of the working population is likely to lead to an increase in the prevalence of chronic diseases. To create possibilities for retirement pensions and health care for the growing population of older people and to compensate for the decrease of young people available for the labour force, policies are being developed to prolong work participation and prevent early exit from the workforce [1]. However, since the prevalence of chronic diseases increases after the age of 45 years [2, 3], the prevalence of chronic disease in older workers is high [4, 5]. Chronic health conditions negatively impact the ability of older workers to stay at work as they are associated with at work productivity loss, and decreased work ability [6–8]. To improve participation in paid work in the large population of older individuals with chronic diseases, it is necessary to first understand what factors affect work participation. Previous work has either focused on predictors of participation in paid work within specific patient populations with one chronic disease or on populations with any chronic disease without differentiating between specific diseases [9, 10]. Rytsala [9] concluded that higher age, lower level of functioning, and a longer duration of depressive episodes were predictors for long term work disability in people with major depressive disorder. Ropponen et al. [10] showed that physically heavy work was predictive for work disability due to low back pain in a Finnish population study.

There are some indications that generic factors for work participation exist. Baanders et al. [7] concluded that generic factors (e.g., general perceived health, pain, fatigue, functional limitations and autonomy) were more important than disease specific factors to predict participation in paid work among patients with a chronic disease. Koolhaas et al. [11] conducted a qualitative study and concluded that there were no major differences between the types of problems encountered by older workers with and without chronic disease.

So far, no study has focused on investigating differences and similarities between different chronic diseases regarding factors important for work participation. However, the factors that are important for work participation in a heterogeneous population of workers with different chronic diseases might be attributable to the largest subgroup in the sample with different chronic diseases, rather than to the existence of a generic predictor. Alternatively, factors may not be discovered in analyses because of the heterogeneity of the sample. Both situations may lead to biased conclusions.

A recent qualitative study contributed to the understanding of factors important for work participation by illustrating how different pathways relating to an (im)balance between work demands and personal resources exist through which poor health could influence work productivity [12]. By applying a mixed method design, qualitative research and quantitative research give complementary information about factors important for participation in paid work. This may increase our insight into which factors are similar and which factors differ among disease groups.

Therefore, the aim of this study was to gain insight into differences and similarities between factors important for participation in paid work in individuals with different chronic diseases by applying a mixed method approach. To gain insight into these differences and similarities, we aimed to include diseases that provided a contrast of symptoms (physical vs. psychological), prognosis (progressive vs. stable), availability of treatment, and risk of dying (Table 1). The three common diseases that were chosen to offer these contrasts were depression (D), cardiovascular disease (C), and osteoarthritis (O).

**Table 1:** Distinct differences between the three disease groups by their main characteristics

Characteristics disease	Nature of symptoms	Prognosis	Treatment available?	Risk of dying?
Depression	Psychological	Recurrences	Yes	Yes
Osteoarthritis	Physical	Progressive	No	No
Cardiovascular disease	Psychological and physical	Progressive/recurrences	Yes	Yes

## Methods

### Design

An integrative sequential design was applied with a mixed method approach. This implies that both quantitative and qualitative methods were integrated to answer the research question. We worked sequentially, as we started with the quantitative analyses, followed by the qualitative analyses, which were followed by another quantitative analyses. This way, the information retrieved from both methods is allowing exchange of information between both methods [13–15].

### Mixed Method Approach

The aim of this mixed method approach was complementarity [13–15]. The complementarity was aimed for because it was expected that the worker perspective in the qualitative part would be complementary to the quantitative part, which relied on questionnaires developed and selected by research professionals rather than by lay persons or patients. First, quantitative data were analysed. The quantitative results were used as input for the topic list of the qualitative study, consisting of in-depth interviews. Next, in-depth interviews were held. Based on the results of these in-depth interviews, new quantitative data analyses were performed as new ideas for potential predictors came up (e.g., mastery was not included in the first analyses, but was added when after the first discussion of the qualitative results). Finally, the interview results and quantitative analyses were used to assess the level of saturation and to decide about adding additional interviews and about the focus of these interviews (e.g., after the first analysis, it became clear that a male with osteoarthritis that was no longer working was missing in our sample. Based on which it was decided to add another interview with a person from this category) [16].

### Quantitative Method

#### Sample

**Fig. 1:**
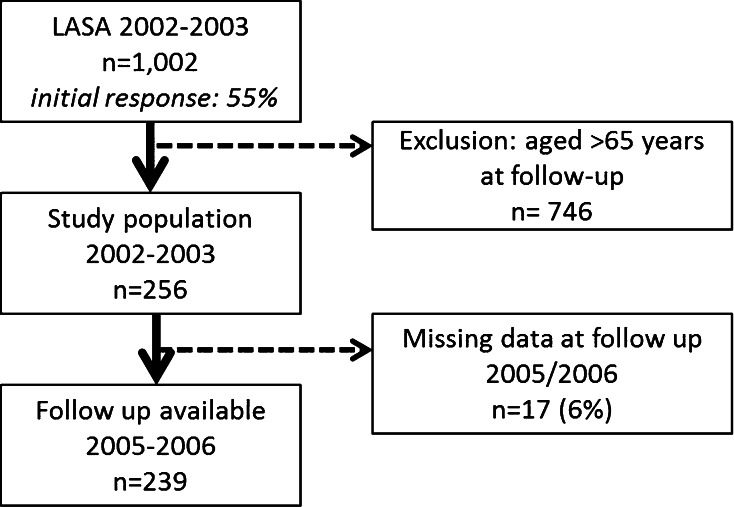
Flow diagram of quantitative part

For the quantitative analyses we used data from the Longitudinal Aging Study Amsterdam (LASA), an ongoing multidisciplinary cohort study focusing on predictors and consequences of changes in well-being and autonomy in the older population. In 2002–2003, a sample of 1002 respondents was recruited (aged 55–65 years; initial response rate 55 %) [17]. The flow of participants for the present study are presented in Fig. 1. All measurements, for the present study consisting of questionnaires, were performed by trained interviewers who visited the participants at home. Details on the sampling and data collection procedures have been described elsewhere [17]. The Medical Ethics Committee of the VU University Medical Center approved of the LASA study; informed consent was obtained from all respondents.

All respondents who were younger than 65 years at the time of the follow-up interview in 2005–2006 and who had D, C or O, or a combination of at least two of the three disorders (DCO) and who had valid data on work status in both 2002/2003 and 2005/2006 were selected (n = 239). We assessed all variables included in the quantitative analyses and no relevant differences were observed between those lost to follow-up and those who completed the follow-up measurement in 2005–2006 (93 %) (*p* > 0.05).

#### Measures

*Depression* was defined as a score of at least 16 points on the Center for Epidemiological Studies Depression scale (CES-D). This questionnaire has been shown to have good criterion validity in this population [18]. A score of 16 or higher is the generally accepted cut-off score for a clinically relevant level of depressive symptoms [19]. The *presence of cardiovascular disease* or *osteoarthritis* was determined by single questions about current or previous cardiac disease or peripheral atherosclerosis, or osteoarthritis. Compared to records of the participants’ general practitioners, the agreement between self-reports and medical records proved satisfactory [20].

Our outcome measure was *having paid workat follow*-*up (2005*–*2006)*. Since we expected the largest contrast between those involved in any paid work and those who were not at all involved in paid work, we defined having paid work as working at least 1 h per week at follow-up.

*Socio*-*demographic* variables included gender (male/female), age, highest level of education completed (lower vocational/at least intermediate vocational education), having a partner (yes/no), having a partner with paid work (yes/no), satisfaction with income level (satisfied/not satisfied), and satisfaction with living standard with this income (satisfied/not satisfied).

*Self*-*rated health* was assessed using the question: “How is your health in general?” Response categories were: (1) very good, (2) good, (3) fair, (4) sometimes good, sometimes poor, and (5) poor [18]. This variable was dichotomised into ‘good or very good health’ (yes/no).

*Functional limitations* were assessed using six self-report items pertaining to mobility activities in daily life. The questions were derived from the Organisation for Economic Co-operation and Development (OECD) questionnaire [21, 22], which was translated to Dutch and validated by Statistics Netherlands [23]. This scale was dichotomised into ‘no functional limitations’ (yes/no).

D, C and O were not taken into account for the comorbidity variable in this study as our groups consisted of participants with D, C, O or any combination of D, C or O. *Comorbidity* was assessed by the questions about the presence of chronic non-specific lung disease (asthma, chronic bronchitis, or pulmonary emphysema), cardiac disease, peripheral atherosclerosis, stroke, diabetes mellitus, rheumatoid arthritis, or cancer.

*Mastery* was measured using a shortened version of the Pearlin Mastery Scale [24], which consists of five negative items, with categories ranging from 1 (strongly agree) to 5(strongly disagree). The score ranges from 5 to 25, such that a higher rating indicates more feelings of mastery in a continuous scale. Mastery is defined as “the extent to which a person perceives himself or herself to be in control of events and ongoing situations”.

*Self*-*esteem* was measured using an adapted version of the Rosenberg self-esteem scale [25], consisting of four items, with categories ranging from 1 (strongly agree) to 5 (strongly disagree). A higher total score (range 4–20) indicates higher self-esteem.

*Neuroticism* and Social inadequacy were measured using a 15-item neuroticism scale and a 10-item social inadequacy scale derived from the Dutch Personality Questionnaire (DPQ) [26, 27].

*Work exposure* was measured by three variables: physical work demands, psychosocial work demands, and psychosocial resources at work, in line with the Job-Demand-Resources model [28]. These work exposure data were derived from a job-exposure matrix, in which occupational classes of the Netherlands Standard Classification of Occupations 1992 (NSCO92) were categorised into the level of probability of exposure to work demands and resources using a Job Exposure Matrix based on data from the Netherlands Working Conditions Survey [29]:

Physical work demands were categorised into: (1) a high probability of exposure to moderate to high physical demands (use of force, uncomfortable work or exposure to repetitive movements) compared to (0) a low probability of exposure to moderate to high physical demands.

Psychosocial work demands were categorised into: (1) a moderate to high probability of exposure to moderate psychosocial demands (task requirements, time pressure or cognitive demands) compared to (0) a low probability of exposure to moderate psychosocial demands.

Psychosocial resources at work were categorised into: (1) a high probability of low resources (low autonomy, low task variation, low supervisor support or low co-worker support) compared to (0) a low probability of low resources.

To enhance readability of this paper, these three variables will be referred to as physical demands (high/low); psychosocial demands (high/low) and psychosocial resources (high/low).

In addition, the *number of working hours per week* was investigated. The latter is important as in the Netherlands, part time work is very common, in particular among women [30].

#### Analyses

Participants were classified into one of four mutually exclusive groups: D, C, O, and DCO. The DCO group consist of participants with either D&O, D&C, O&C or D, C and O. *t* tests and Chi square tests were performed to study differences among the four groups. Then the homogeneity of each predictor across the four groups was assessed considering the approach proposed by Dyer as follows [31, 32]: To examine similarities and differences between predictors of work participation among the four disease groups, descriptive analyses were performed for each of the potential predictors separately for the four groups by *t* tests and Chi square tests. The homogeneity of each predictor for having paid work at follow-up across the four disease groups was assessed using the approach proposed by Dyer [31, 32] as follows:

- For each potential predictor a logistic regression model was fitted with having paid work in 2005–2006 as dependent variable, separately for the four groups (D, C, O, DCO);
- a pooled estimate was computed by weighing both coefficients. Weighing was performed by dividing each coefficient by its variance, and then summing over the weighed coefficients. The pooled estimate was then calculated by dividing the sum of the weighed coefficients by the sum of the inverse of the variance of the coefficients;
- the pooled estimate was used in a Chi square-test for coefficients to verify the null hypothesis that the coefficients in both groups were equal and did not significantly differ from the pooled estimate (i.e., whether the predictor was homogeneously distributed across groups) (Χ^2^ < 7.8; *DF* = 3);
- if the hypothesis of homogeneity was not rejected, the pooled estimate from (b) was examined and the significance of its association with the outcome was tested using a *t* test [31].
- if the hypothesis of homogeneity was rejected, the coefficients in each group stratum were presented and compared using a *t* test for estimated coefficients [33] including corrections for multiple comparisons with the Bonferroni test [34]

For gender, level of education, functional limitations, comorbidity, and having a partner with paid work, we could not test differences among the four groups because for the groups with depression, there was not equal distribution over the 2 × 2 table (e.g., male work/male no work/female work/female no work) leading to empty cells.

### Qualitative Method

The qualitative part was performed by CB, AdK and TA. The results were discussed by the whole author team.

#### Sample

Semi structured in-depth interviews were held in 2011 and 2012. Emergent sampling to select a heterogeneous group of respondents based on gender, work status and disease (D, C, or O). Emergent sampling implies that our study population emerges, and unfolds as the study progress rather than that it is constructed prior to the study. Following this, we started the recruitment, and based on the characteristics of the first participants (working/not working, male/female, D/C/O), it was decided which specific participants should be added and recruited for the final interviews [35]. Participants were recruited through patient organizations, collectives by e-mails, or posts on patient websites. Inclusion criteria were aged between 45 and 65 years, involved in paid work at present or maximum 5 years ago.

A total of fourteen interviews were conducted. The sampling stopped when no new information came up during the interviews, which implies that data saturation had been reached [16, 36].

#### Interviews

A semi-structured interview-guide, including a topic list, was developed from the quantitative part of this study and previous research. It facilitated discussion of a range of factors that could potentially have helped or hindered functioning in paid work, such as health complaints, work characteristics, personal situation, social support at home or at work, or financial issues. The in-depth interviews lasted 60–90 min. In-depth interviewing is defined as a conversation with a specific research purpose, and focuses on the informant’s perception of self, life and experience, expressed in his or her own words. It allows the researchers to understand the particular and private interpretations of social reality that individuals hold [37, 38]. Two experienced interviewers held the interviews. Due to external circumstances, the first interviewer conducted the first two interviews only, after which the second interviewer conducted the other interviews. The main question of the interviews was ‘Why are you still working?’ and ‘Why have you stopped working?’ for respondents who were still working and who stopped working respectively. The interviews were conducted in the respondents’ homes, and interviews were recorded and transcribed verbatim. Before starting the interview, informed consent was obtained, including consent for audio taping the interview. To reduce bias and ensure validity a member check was carried out: every respondent received a report of the interview to check for accuracy of interpretation [39].

#### Analyses

All interviews were analysed using thematic content analysis based on comparisons within and across respondents. Data analysis of the first interview was done by two researchers (of which one was the interviewer) so that they could agree upon a method of coding. The analyses of the remaining interviews were performed by the most experienced qualitative researcher who also conducted most of the interviews and consisted of three steps [16, 36].

First, the transcripts were read several times. The texts were divided into fragments, and codes (labels) were assigned to these fragments (open coding). Subsequently, codes were assigned to themes and finally, the categories of the several transcripts were related to one another (axial coding) [16, 36]. These codes were all organized into a mind map, using the computer program Mindjet Mindmanager. The preliminary conclusions based on this mind map were thoroughly discussed in the project team, based on which codes could be reformulated or ordered differently.

The last phase of the analysis was selective coding. This implies that the essence of what each theme was about was identified, searched for relations through constant comparison across cases (individual interviews), looked for deviant cases, and analysed variation within and between cases. Finally the different themes were fit into the broader overall ‘story’ that the data told us, to gain insight into differences and similarities among D, C and O and to investigate differences and similarities between the findings from the quantitative and qualitative analyses.

All findings were discussed in the whole project team twice, once in a preliminary stage to discuss the codes, and once to discuss the interrelations between the codes to reach the main findings. During these discussions, ideas for additional quantitative predictors were gathered based on which we performed additional quantitative analyses.

## Results

### Sample Characteristics

In the quantitative part of this study, C consisted of more males (64 %), whereas women were predominant in D (66 %) and O (72 %) (Table 2). At baseline, the group with DCO was least often involved in paid work (26 %), followed by D (40 %). At follow-up, C and O showed the highest participation rates in paid work (44 and 34 %, respectively) versus the other two groups (<20 %).

**Table 2:** Descriptive characteristics of four mutually exclusive disease groups: depression (D), cardiovascular disease (C), osteoarthritis (O) or any combination of D, C and/or O (DCO)

Population characteristics	D (n = 35)	C (n = 44)	O (n = 120)	DCO (n = 57)
Female gender, n (%)	23 (66)	16 (36)	74 (62)	36 (63)
Age (2002/2003) (years), mean (SD)	58.2 (2.1)	58.4 (2.2)	58.4 (1.9)	58.2 (2.1)
Intermediate or higher education, n (%)	21 (60)	22 (50)	66 (55)	28 (49)
Having a partner, n (%)	27 (77)	40 (91)	109 (91)	44 (64)
Having a partner with paid work, n (%)	15 (58)	16 (44)	48 (46)	14 (33)
Very good or good self-rated health, n (%)	17 (49)	22 (50)	68 (57)	14 (24)
No functional limitations, n (%)	17 (49)	35 (80)	73 (61)	20 (36)
Comorbidity^a^, n (%)	28 (80)	26 (59)	77 (64)	43 (75)
Neuroticism (0–30), mean (SD)	10.6 (7.1)	5.5 (5.2)	6.0 (5.0)	12.0 (7.6)
Social inadequacy (0–20), mean (SD)	6.7 (4.9)	4.5 (4.4)	5.7 (5.0)	7.8 (5.5)
Mastery (5–25), mean (SD)	15.7 (3.4)	18.6 (3.1)	18.4 (2.9)	14.7 (3.9)
Self-esteem (5–20), mean (SD)	13.7 (2.6)	15.8 (2.2)	15.4 (2.0)	13.1 (2.7)
Satisfied with income level, n (%)	22 (63)	24 (56)	84 (70)	27 (47)
Satisfied with living standard, n (%)	21 (60)	30 (70)	88 (73)	25 (44)
Subgroup with paid work at baseline (2002/2003)*, n (%)	14 (40)	24 (55)	60 (50)	15 (26)
Occupational skills, mean (SD)	2.9 (1.0)	2.9 (0.9)	2.8 (1.2)	3.1 (1.0)
Occupational prestige level, mean (SD)	42.6 (14.6)	41.5 (13.3)	39.3 (16.4)	42.2 (17.0)
Number of hours work/week, mean (SD)	24.9 (12.1)	30.2 (16.9)	30.0 (15.7)	26.1 (16.8)
High physical work demands, n (%)	10 (83)	15 (71)	43 (75)	8 (53)
High psychosocial work demands, n (%)	4 (11)	6 (14)	22 (18)	7 (12)
Low psychosocial resources at work, n (%)	11 (31)	18 (41)	52 (43)	13 (23)
Response in 2005–2006, n (%)	33 (94)	43 (98)	112 (93)	55 (96)
Having paid work (2005–2006), n (%)	6 (18)	19 (44)	38 (34)	9 (16)

^a^This involves all comorbidities other than D, C, O

* Some variables are only available for the group having paid work at baseline

In the qualitative part of this study, the participants were aged 47–64 years and gender and work status varied within each disease group (Table 3). An exception on this is D, where all participants were at work at the time of the interview. However, they all had experienced periods of unemployment or absence from work in the past and one was on long term sick leave during the interview.

**Table 3:** Descriptive characteristics of participants with depression (D), cardiovascular disease (C) and/or osteoarthritis (O) in the qualitative study

Characteristics	D (n = 4)	C (n = 5)	O (n = 5)
Gender male/female	1/3	4/1	2/3
Age (years)	47–57	55–63	56–64
At work yes/no	4/0	3/2	2/3
Duration of exit from paid work	All working at present	2–4 years	2 months–4 years

### Factors Important for Participation in Paid Work for D, C, O, and DCO

The stratified quantitative analyses are listed in Table 4. As group sizes are small, the results should be interpreted with caution. In general, effect sizes are either small, and point in similar directions. For the remainder of the manuscript, we will focus on the effects of the pooled estimates for the quantitative analyses (Table 5).

**Table 4:** Stratified analyses of factors associated with work participation in participants with depression (D), cardiovascular disease (C), osteoarthritis (O) or any combination of D, C and O (DCO)

Predictors		D (n = 35)	C (n = 44)	O (n = 120)	DCO (n = 57)	Predictors		D (n = 35)	C (n = 44)	O (n = 120)	DCO (n = 57)
Female gender	B	*	−1.16	−1.75	−0.92	No functional limitations	B	*	−1.25	−0.63	−0.08
	SE		0.70	0.44	0.75		SE		0.87	0.43	0.80
	HR		0.31	0.17	0.40		HR		0.29	0.53	0.93
Age (years)	B	0.24	−0.32	−0.31	−0.09	Very good or good self rated health	B	0.92	0.65	0.77	0.94
	SE	0.23	0.11	0.43	−0.22		SE	0.95	0.62	0.42	0.76
	HR	1.26	1.11	1.53	0.80		HR	2.50	1.92	2.17	2.56
Intermediate or higher education	B	*	0.11	0.43	−0.22	Comorbidity	B	*	−0.99	−0.31	−1.22
	SE		0.61	0.41	0.74		SE		0.64	0.42	0.78
	HR		1.11	1.53	0.80		HR		0.37	0.74	0.29
Having a partner	B	−0.56	−0.26	−0.17	−0.82	Neuroticism	B	−0.15	−0.02	−0.14	−0.09
	SE	0.98	1.05	0.76	0.75		SE	0.10	0.06	0.05	0.06
	HR	0.57	0.77	0.85	0.44		HR	0.12	0.98	0.87	0.92
Paid job (2002/2003)	B	2.74	3.99	3.07	3.25	Social inadequacy	B	−0.28	0.02	−0.09	−0.07
	SE	1.17	1.13	0.59	0.93		SE	0.86	0.07	0.04	0.08
	HR	15.60	54.00	21.46	25.90		HR	0.11	1.02	0.92	0.93
Partner with paid work (2002–2003)	B	*	2.11	1.70	−0.05	Mastery	B	0.06	0.08	0.22	0.08
	SE		0.78	0.47	0.94		SE	0.14	0.10	0.09	0.10
	HR		8.25	5.45	0.95		HR	1.06	1.08	1.24	1.08
Satisfied with income level	B	1.39	0.63	0.50	−0.13	Self esteem	B	−0.10	0.11	0.15	0.33
	SE	1.16	0.63	0.45	0.74		SE	0.16	0.15	0.11	0.19
	HR	4.00	1.87	1.64	0.88		HR	0.90	1.12	1.16	1.40
Satisfied with income and living standard	B	1.54	0.88	0.31	0.61						
	SE	1.16	0.71	0.46	0.74						
	HR	4.64	2.41	1.36	1.84						

Predictors		D (n = 13)	C (n = 24)	O (n = 55)	DCO (n = 12)	Predictors		D (n = 13)	C (n = 24)	O (n = 55)	DCO (n = 12)
Subgroup with paid work at baseline only											
Number of hours work per week	B	−0.07	0.02	0.04	0.00	High physical work demands	B	−0.69	−20.80	−1.41	1.67
	SE	0.06	0.03	0.02	0.04		SE	1.58	16,409	0.84	1.35
	HR	0.93	1.02	1.04	1.00		HR	0.50	0.00	0.24	5.33
Occupational skills level	B	0.14	0.41	0.47	−1.05	High psychosocial work demands	B	0,69	0.65	0.09	−22.12
	SE	0.63	0.55	0.28	0.78		SE	1,22	1.22	0.59	17,975
	HR	1.16	1.51	1.60	0.35		HR	2,00	1.92	1.09	0.00
Occupational prestige level	B	−0.01	0.06	0.04	−0.08	Low psychosocial resources at work	B	−1.54	−20.51	0.22	0.41
	SE	0.04	0.04	0.02	0.05		SE	1.41	16,409	0.82	1.55
	HR	0.99	1.06	1.04	0.92		HR	0.21	0.00	1.25	1.50

**Table 5:** Pooled estimates, X^2^ tests for homogeneity for all multivariate coefficients; *t* test on pooled estimates for homogeneous variables to assess the association with having paid work in 2005/2006

	Pooled estimate	X^2^ test *H*0: *X* ^2^ < 7.8 (*DF* = 3)	*t* test *H*0:-1.96 < *T*<1.96	Odds ratio	95 % CI
Female gender^a^	−1.45	1.16	−**4.39**	**0.23**	**0.12**–**0.45**
Age (years)	−0.21	5.68	−**2.71**	**0.81**	**0.70**–**0.94**
Intermediate or higher education	0.32	1.83	1.07	Ns	
Having a partner	−0.43	0.32	−0.99	Ns	
Partner with paid work^a^	1.49	3.58	**4.19**	**4.42**	**3.70**–**15.77**
Financial economic variables					
Paid work in 2002/2003	3.16	0.91	**7.44**	**23.52**	**10.61**–**56.01**
Satisfied with income level	0.48	1.34	1.51	Ns	
Satisfied with income and living standard	0.59	1.20	1.80	Ns	
Functional limitations					
No functional limitations^a^	−0.62	0.99	−1.80	Ns	
Health					
Very good or good self-rated health	0.79	0.10	**2.62**	**2.19**	**1.22**–**3.95**
Comorbidity^b^	−0.63	1.51	−**1.98**	**0.53**	**0.29**–**0.99**
Personality					
Neuroticism	−0.10	2.57	−**3.11**	**0.91**	**0.85**–**0.96**
Social inadequacy	−0.07	3.26	−**2.16**	**0.93**	**0.88**–**0.99**
Mastery	0.13	1.88	**2.49**	**1.13**	**1.03**–**1.25**
Self esteem	0.12	3.28	1.69	1.12	0.98–1.29
Workers at baseline only					
Number of hours work per week	0.02	3.43	1.49	Ns	
Occupational skills level	0.30	3.48	1.35	Ns	
Occupational prestige level	0.03	6.22	1.55	Ns	
High physical work demands^a^	−0.58	1.58	−0.89	Ns	
High psychosocial work demands^a^	0.27	0.32	0.56	Ns	
Low psychosocial resources at work^a^	−0.12	1.31	−0.18	Ns	

Bold values are statistically significant *p* < 0.05

*Ns* not significant

^a^The pooled estimate was calculated for three instead of four groups due to (nearly) empty cells. The corresponding Chi square value for *DF* = 2 is 5.9

^b^Different from the group with depression, cardiovascular disease or osteoarthritis

#### Demographic Variables

For the total group, male gender, lower age, paid work at baseline, and having a partner with paid work were associated with having paid work at follow-up (Table 5). Level of education, having a partner, and satisfaction with income level were not predictive for work status at follow-up.

No significant differences were found for predictors of paid work among participants with D, C, O, or DCO (Table 5). From the qualitative analyses, no demographic characteristics emerged as factors associated with work status.

#### Health-Related Characteristics

Participants with a higher scores on self-rated health (better self-rated health) and without comorbidity were more often involved in paid work at follow-up (Table 5). Lower scores on neuroticism (less neuroticism) and social inadequacy (less social inadequacy) were predictive for paid work at follow-up, whereas self-esteem, the presence of functional limitations and comorbidity were not predictive for work status (Table 5). Mastery was found to be important in both the quantitative part expressed by higher mastery scores (OR 1.13; 95 % CI 1.03–1.25; Table 5) and the qualitative part:

“I can work, I want to work, for sure, and my disease is not really a limitation to work. I can only be my own limitation to work, in my head, when I do recognize my limits, when I do too much, when I start doing things I should not do.” (Cardiovascular disease; woman, 62 years old)

“Well, yes, and I try to eat healthy, and of course not to load (my joints) like an idiot, so you do things in a way to make sure you can continue as long as possible.” (Osteoarthritis; woman, 56 years old)

#### Work Characteristics

In the quantitative analyses, work characteristics were not predictive for work status at follow-up (Table 5). Complementary to this, interviewees explained that the concern and understanding of supervisor and colleagues at the workplace, which can be considered an aspect of psychosocial resources at work, was important:

“I have not produced much over there, but no one was watching me anyway, so there were days I could not really accomplish much. But uhm, well, I have told them, also my supervisor, and he understood it.” (Depression; woman, 55 years)

Work adjustments and autonomy during work e.g., to take a break were mentioned as well:

“If I am a little tired, I will need to take a short break. I just need to do things a little differently and I want to have some buffer to compensate for that.” (Osteoarthritis; woman, 64 years)

“I was 100 % work disabled, and I was having a lot of troubles with my back, and uhm, well, from my back in particular, and uhm, then I searched for work adjustments, for an adjusted work place, together with the occupational physician.” (Osteoarthritis; man, 56 years old)

#### The Importance of Work

The importance of being able to work was not included in the quantitative part of the study. In the qualitative part of the study, the importance of work came up in most interviews, although in different ways. The importance of work for social relationships, income and purpose in life was mentioned by all three disease groups.

“I need to work to help my family; with my salary I can support 10 family members” (Cardiovascular disease; woman, 49 years)

“More the feeling of being part of it [work]. I enjoy it [work], well, it is nice, when you have the feeling, while walking around, that colleagues are having fun because of me, or with me, than I feel I contribute, that feeling, that is important.” (Depression; man, 48 years)

“(…) and to be part of the chain, that you will be missed when you are not there.” (Cardiovascular disease; woman, 49 years)

In addition, work as distraction from worries was mentioned in several interviews:

“Work is very important for me. (…) otherwise I would have been worried about my heart every day. Now I can say, come on, stop worrying and go to work.” (Cardiovascular disease; man, 56 years)

However, only for the participants with depression, the structure provided by work was reported as important as they needed this to cope with their disease. Work was considered as a necessary part of their lives.

“I needed to hold on, continue work, and imagine what if I would lose my job. My job was my basis, it was my identity, really important” (Depression; man, 57 years)

“Work is my primary need in life” (Depression; woman, 47 years)

“The most, most, very most important thing is, to me, as I suffer from depressive symptoms, negative thoughts, etcetera, to distract my senses. Because the moment I am busy with something, well, than I do not have time to worry about things, because other things request my attention.” (Depression; woman, 56 years)

#### Work Adjustments

In the interviews, adjustments in work were important for all three groups, albeit in a different way.

“No. Well, my boss considered me, how shall I put this, a nuisance. Because it was a boss that ignored rules and such, and yes, I explained my rights, and he just told me it was not gonna happen, whether I liked it or not. Nothing I could do about it.” (Osteoarthritis; man, 58 years)

Participants with osteoarthritis who continued working indicated that work adjustments were a complicated issue. Those who were able to make their own arrangements had to be careful in explaining this, as others might consider this as selfish behaviour. Those who were unable to make their own arrangements mentioned that it had not been easy to accomplish a work adjustment. This was different for the participants with depression and cardiovascular disease, who had more positive perceptions of the effectiveness of work adjustments.

#### Work Participation

During the interviews, return to work came up as an important theme in the context of work participation. Some differences among the disease groups were found regarding factors associated with return to work. Participants with osteoarthritis seemed to stay out of work once they had left work. They would prefer to stay at home if they had a choice, financially speaking. Another reason for not working was that they felt insecure about their work, and had lost their faith to be treated fairly. Because of this, they chose to take back control over the situation and decided to quit working altogether.

“So, actually, it would be great to stay at home with him (retired husband), but well, that is financially not really feasible” (Osteoarthritis; woman, 55 years)

“Well uhmm, the way the law (for work disability) is constructed, it’s very unfair” (Osteoarthritis; man, 58 years)

Participants with depression mentioned the difficulties they had to maintain their job, in particular in a temporary employment contract with a continuous necessity to perform at their best which they considered as an additional challenge. Support from the supervisor was mentioned as an important factor for return to work in the group with depression; they perceived support of their supervisor as long as they performed well, which again increased their drive to perform at their best.

“Well, I felt I had to perform again, that I had to show again… so there I went again, with a bucket full of stress.” (Depression; woman, 47 years)

“It’s a great employer, and my director supported me a lot, although she is really busy, but I know she wanted to give me this chance.” (Depression; woman, 59 years)

## Discussion

The aim of this mixed methods study was to gain insight into differences and similarities in factors important for work participation among older workers with three different chronic diseases: depression (D), cardiovascular disease (C), and osteoarthritis (O). Most factors important for participation in paid work were similar for D, C and O. However, the qualitative part of our mixed methods study complemented our quantitative findings as it showed that the meaning of these factors for the group with depression differed from the other disease groups. Moreover, work characteristics were not predictive for work participation in the quantitative analyses, but the interviews showed that the way participants managed their work was important for work participation.

### Factors Associated with Participation in Paid Work with D, C, and O

Self-perceived health was a predictor of work status in all groups in the quantitative analyses; participants with better self-perceived health and no comorbidity were more likely to be involved in paid work. In a systematic review on prognostic factors for work disability, perceiving more health complaints were predictive for work disability in patients with chronic somatic disease, including arthritis [40].

Knowing one’s limits and balancing energy were considered important aspects continuing paid work. A qualitative study by Leijten et al. [12] showed that the influence of health on productivity was the result of an imbalance between an individual’s resources at work and the health problem. This study supports our findings as mastery was found to be important for having paid work at follow-up both in the quantitative and qualitative study.

The main difference among D, C and O we observed was found for workers with depression. Even though the importance of work was mentioned as important for work participation by all disease groups, the meaning differed. Only workers with depression mentioned a sense of urgency to work, as work provided structure to their day, which is an important element of the treatment of depression.

An additional challenge for workers with depression might be that arranging work adjustments can be more difficult, as their disease may interfere with the motivation to make such arrangements, or the initiative needed to discuss limitations at work with the supervisor. Not discussing the need for work adjustments may pose these workers at risk for dropping out, as previous research has shown that implementing work adjustments was associated with positive effects on functioning in work in workers with chronic disease [41].

Although in the quantitative analyses, no differences in predictors were found among the three chronic diseases, in some cases we were unable to test differences for the group with depression. The reason for this was that all workers with depression had similar scores for certain predictors, which made it impossible to perform Chi square tests. The descriptive results show that participants with depression have worse outcomes on all potential predictors compared to the other groups. This is in line with an earlier study where it was shown that common mental health problems had a larger impact on productivity at work than physical health problems [42].

### Methodological Considerations

The major strength of this study is its mixed method design. We performed quantitative analyses on a representative prospective dataset [17], which we complemented with interview data. As the quantitative data were collected between 2002 and 2006, we decided not to interview this population as the risk for recall bias was expected to be too high as they potentially had quit working more than 10 years ago. Over the past years, policy measures have been taken to prolong work participation of older workers. Although we do expect that older workers are increasingly encouraged to prolong their working life leading to retirement at a higher age, we do not expect that this has affected participants with D, C or O differently.

An important difference between the populations involved in the quantitative and qualitative studies is that for the latter, the interviewees were all recruited through patient-oriented channels (such as patient organisations). These participants are more likely to be actively involved in their disease process and because of this may not be fully representative.

The participants in the quantitative pare were assigned based using two different methods: self reports (O and C), and a depression scale for D. The self report questionnaire we used have been validated within the LASA population [20]. The group D in the quantitative part was identified based on questionnaire scores on a depression scale instead of self-reports which has been found to be reliable to predict depression in this population [18]. It is therefore unknown if this group was aware of their depression disorder. In addition, group assignment based on a scale differs from assignment based on self-report, as was the case for the groups with osteoarthritis and cardiovascular disease. This difference between group assignment may have led to differences between groups. However, we do not expect that this has biased the associations between work factors and work participation. A limitation of the quantitative part of our study is the relatively small sample available for the analyses. Because of this, it was not possible to correct for confounders in any of the analyses. However, since this is the first study to undertake these analyses, the results can be considered as a first necessary step towards understanding similarities and differences between factors associated with participation in paid work among individuals with different chronic diseases. Another limitation of this study is that the sample size of our study did not allow a differentiation between reasons for not participating in paid work. From previous research we know that differences exist between work disability or unemployment as cause for not working [7]. Future research should take the reason for not working into account when possible. For the qualitative part of our study we used emergent sampling. This enabled us to aim for purposeful sampling without losing flexibility in challenging recruitment conditions. We started interviewing volunteers, and checked the variation in our sample throughout the process, based on which we searched for additional volunteers with specific characteristics. This way we managed to include a rather heterogeneous group of participants, with males and females of different ages. More importantly, we reached saturation which supports the trustworthiness of our findings.

### Implications for Research and Practice

The most important research implication of this study is that for the investigation of factors associated with participation in paid work, mixed method designs should be considered, as complementary information is revealed. Qualitative research can be considered of additional value to quantitative research by providing in depth knowledge about how factors identified in quantitative studies may influence work participation. Factors related to work characteristics and health that were similar for different chronic diseases in the context of work participation offer opportunities for intervention development and policy measures as these can be targeted to the larger population of older individuals with any chronic disease rather than on relatively small patient populations with a specific disease. However, the differences observed between depression and the other two diseases, such as the meaning of work, deserve more attention. In addition, interventions may be implemented to enhance mastery to maintain work participation with a chronic disease at older age. Additionally, supervisors and colleagues may play a role in lowering the threshold at work to ask for work adjustments. Taking into account the social context may give way to different strategies to enhance working life, as a working partner facilitates continuing work. Future research could focus on exploring the mechanisms of the social context relating to prolonging work participation, such as e.g., how involvement in paid work of the partner influences work participation.

## Conclusion

Many factors important for work participation were similar for D, C and O. However, the interviews revealed that for D, the context and the meaning attributed to these factors differed. The qualitative part was complementary as we retrieved information about the context and meaning of predictors, and gave rise to new factors to be considered in future research that were not taken into account before.
